# Neuroelectric Evidence for Cognitive Association Formation: An Event-Related Potential Investigation

**DOI:** 10.1371/journal.pone.0034856

**Published:** 2012-04-19

**Authors:** Alice S. N. Kim, Malcolm A. Binns, Claude Alain

**Affiliations:** 1 Rotman Research Institute of Baycrest, Toronto, Ontario, Canada; 2 Department of Psychology, University of Toronto, Toronto, Ontario, Canada; 3 Dalla Lana School of Public Health, University of Toronto, Toronto, Ontario, Canada; City of Hope, United States of America

## Abstract

Although many types of learning require associations to be formed, little is known about the brain mechanisms engaged in association formation. In the present study, we measured event-related potentials (ERPs) while participants studied pairs of semantically related words, with each word of a pair presented sequentially. To narrow in on the associative component of the signal, the ERP difference between the first and second words of a pair (Word2-Word1) was derived separately for subsequently recalled and subsequently not-recalled pairs. When the resulting difference waveforms were contrasted, a parietal positivity was observed for subsequently recalled pairs around 460 ms after the word presentation onset, followed by a positive slow wave that lasted until around 845 ms. Together these results suggest that associations formed between semantically related words are correlated with a specific neural signature that is reflected in scalp recordings over the parietal region.

## Introduction

Cognitive theorists have made a qualitative distinction between memory for individual items and for associative information [1]. Item information corresponds to individual items or events, such as the presentation of a single word. Associative information, on the other hand, corresponds to links between items, such as the presentation of two words as a pair or single unit. Although much work has been done to investigate the neuroelectric correlates of item encoding [2]–[4], what is lacking, to our knowledge, is evidence that the neuroelectric correlates of item encoding and association formation differ. Such evidence would complement behavioral and neuropsychological findings that suggest different memory processes may underlie item and associative memory [5], in addition to the available fMRI evidence [6], [7]. Differentiating the neurolectric correlates of item encoding and association formation would also provide information about the timing of processes that may be differentially engaged in item encoding and association formation. Since many studies have already investigated the neuroelectric correlates of item encoding, in the present study we focused our efforts on examining the neuroelectric correlates of association formation, with a special emphasis on separating out the effects of any processes that support item encoding.

A good deal of evidence about how the brain supports encoding has been revealed through the use of the “subsequent memory paradigm” [8]–[10]. In this paradigm, participants' brain responses are recorded while they are presented with study items (encoding phase). Afterwards, participants are tested on their memory for these study items (test phase). The brain responses that were recorded for each item during the encoding phase are then sorted and analyzed based on whether the given study item was subsequently retrieved on the memory test. This type of analysis allows one to examine differences between encoding phase brain responses recorded for subsequently recalled and subsequently not-recalled study items, as measured by the employed memory test. The differences found in the brain responses are thought to reflect how effectively a memory trace is formed.

Past studies have used the subsequent memory paradigm to show that the amplitude of event-related potentials (ERPs) elicited by to-be-remembered items, during both intentional [11] and incidental [12] memory paradigms, can predict the accuracy of retrieval on a subsequent memory test. The general finding is that ERPs elicited by those items that were subsequently retrieved demonstrated a larger positive deflection compared to the ERPs elicited by items that were subsequently forgotten. This difference in ERPs provides a measure of encoding and has been referred to as ‘Dm’ – difference based on later memory performance [12], an ERP ‘memory effect’ [13] and a ‘subsequent memory effect’ [14]. The subsequent memory effects (SMEs) reported in the ERP literature are composed of three components: the early and late positive components [15] and the slow wave. The early component begins around 250 ms after stimulus onset, and is largest over the frontal region of the scalp compared to the central and parietal scalp regions. The late component begins around 450 ms after stimulus onset and has been investigated much more extensively compared to the early component. Parameters that have been shown to modulate the late SME component include the encoding strategy used [12], whether the memory test was explicit versus implicit [16], the type of stimuli used [17], and the strength of the subsequent memory [18]. The slow wave begins around 500 ms after the stimulus onset and has been associated with elaborative, as opposed to rote, encoding [18], [19].

In the first study that reported encoding-related ERP data in relation to participants' subsequent memory performance, Sanquist and colleagues [20] presented participants with pairs of words to study, with each word of a pair presented sequentially. Participants' task was to judge whether the two words were the same or different based on one of three criteria: orthographic, phonemic, or semantic attributes. Later, the participants were tested on recognition of words that were presented second within a pair, but they were not tested on their memory for associations. Participants' performance on the recognition test for the second word of a pair was used as the basis for the subsequent memory analysis. Semantic comparisons led to the highest percentage of recognized words, followed by the phonemic and then the orthographic comparisons. Subsequently recognized words elicited a larger positive amplitude for the late positive component and slow wave compared to words that were not subsequently recognized. For the semantic condition, the late positive component difference peaked at about 500 ms after the onset of the first and second words of a pair and was largest at the midline parietal scalp region (i.e., Pz). The slow wave difference started approximately one second after the onset of the second word of a pair and appeared larger in the more anterior, compared to posterior, regions of the scalp. A number of other studies have found similar SME in relation to recognition and recall of single words [2], [14], [15].

Here we describe an ERP study that extends the work of past studies on association formation. In the present study, we re-analyzed electroencephalography (EEG) data from an existing database [21]. These data were acquired while participants studied and encoded pairs of semantically related words, with each word of a pair presented one at a time. The EEG data were collected under two conditions that varied in the degree of intra-list semantic similarity. For the purposes of the present study, ERPs from both conditions were combined to increase the overall number of observations of pairs that were subsequently recalled and pairs that were not subsequently recalled. In both of these conditions, each word of a pair belonged to the same semantic category.

The encoding-related EEG data that were re-examined in the present study were originally analyzed on the basis of intra-list semantic similarity and subsequent paired associate recall, the results of which have been reported previously [21]. Here we will highlight the relevant findings. First, subsequently recalled pairs, compared to subsequently not-recalled pairs, demonstrated a larger positive deflection in the ERP waveform around 555 ms after each word of a pair was presented. These positive deflections were interpreted as reflecting the encoding of each individual word. Second, a frontal-positive late wave (LW), which occurred between 1 and 1.6 seconds after the presentation onset of the second word, also demonstrated a larger positive deflection for those pairs that were subsequently recalled. Given the timing of the LW, combined with its amplitude pattern, it was thought to reflect association formation. However, since associations between pairs of words may begin to form prior to the time range of the LW, in the present study we examined whether cognitive association formation would be reflected in the ERPs recorded during the presentation of the second word. To do so, we focused our efforts on differentiating the neuroelectric signal corresponding to the encoding of the second word (item encoding) and the neuroelectric signal corresponding to association formation.

To separate the electrical brain activity corresponding specifically to association formation from that corresponding to item encoding, the present study extended our previous investigation by examining the difference between the encoding-related ERPs to the first and second words of a pair (Word1 and Word2, respectively). Since the ERPs recorded for Word1 reflect the encoding of Word1 and the ERPs recorded for Word2 reflect both the encoding of Word2 and the association formed between the two words of a pair, the ERPs elicited by Word1 and Word2 can be contrasted to differentiate the neuroelectric correlates of association formation and item encoding. To narrow further in on the neuroelectric correlates of association formation, we compared this ERP difference (Word2-Word1) for the pairs that were subsequently recalled and pairs that were not subsequently recalled. This second ERP difference will be referred to as the ‘double difference.’ The double difference waveform was thought to better reflect successful association formation, compared to its constituent waveforms, for the following reason: the Word2-Word1 ERP difference of both the subsequently recalled and subsequently not-recalled pairs likely reflected brain responses related to the sequential presentation of the words of a pair (e.g., habituation), however, the Word1 versus Word2 ERP difference of the subsequently recalled pairs also reflected brain responses underlying association formation. Thus, by contrasting the Word2-Word1 ERP difference of subsequently recalled and subsequently not-recalled pairs, we were able to focus on those ERP components that likely reflected association formation.

Previous studies that have investigated the neuroelectric correlates of association formation [22]–[24] did not differentiate association formation from item encoding, as they either presented both items of a pair together at the same time or simply did not focus on this aspect. For example, Kounios and colleagues [22] conducted an ERP study to determine whether the use of two different associative strategies (compositional versus fused representations) would result in different electrophysiological patterns. In this study, participants were presented with pairs of words, with each word of a pair presented one at a time. Participants were later tested on their memory of the order in which the words of a pair were presented. However, Kounios and colleagues did not report the results of a subsequent memory analysis. Instead, for those pairs that were subsequently remembered, the encoding-related ERP data were analysed in relation to participants' response speed on the memory test. Thus, the encoding data were analyzed to predict participants' subsequent response speed for remembered pairs as opposed to participants' subsequent memory performance.

More recently, in a study by Caplan and colleagues [23], participants were presented with words to study, one word at a time. The words were either grouped into pairs or short lists composed of three words. Later, participants were tested on their memory of the pairs using cued recall, where the target could be probed with the word that was either shown before or after it during the study phase. The corresponding results were used as the basis for the subsequent memory analysis. The study aimed to differentiate the neuroelectric correlates of association formation and serial list learning and both the first and second words of a pair were averaged together. Consequently, the experimental design makes it difficult to separate the neuroelectric correlates of item encoding and association formation.

Our hypotheses were formed on the premise that any ERP component reflecting association formation would occur primarily after the presentation of the second item. Although participants may start preparing to make an association when they are presented with the first item or even earlier, an association between two particular items can only begin to form after the second item is presented. Therefore, we hypothesized that ERP component reflecting association formation would be revealed as amplitude differences between the first and second words of those pairs that were subsequently recalled, and that no such differences would be found for those pairs that were not subsequently recalled. The ERP differences reflecting association formation were expected to occur during the time range of the endogenous P3 wave. Further, in light of previous work demonstrating interdependence between processes engaged in encoding and retrieval [25], combined with findings that the parietal old/new effect reflects recollection of associative information [26], [27], any ERP differences reflecting association formation were expected to occur over the parietal scalp region during the time range of the parietal old/new effect (about 400–800 ms post-stimulus).

## Methods

We have reported the material and methods, including information about the participants and electrophysiological methodology, in our previous paper [21]. Here we summarize it briefly and add a description of the new analyses that were conducted in the present study.

### Ethics Statement

The study was approved by the Baycrest Research Ethics Board and all participants provided written informed consent prior to the experiment.

### Participants

Fourteen healthy, young adults participated in this experiment. All participants had normal or corrected-to-normal vision and no history of neurological or psychiatric disorder. Data from two participants were discarded: one of these participants had too few trials in one of the conditions to allow ERP analysis, and the other participant had large movement artifacts throughout the recordings. As a result, ERP averages were obtained from 12 participants (6 female; mean age: 23 years, range: 19 to 32; first language: English).

### Experimental Procedure

Each participant took part in one experimental session, which consisted of 20 study/test cycles. Each study/test cycle consisted of three phases. During the first phase, participants were presented with a list of 10 pairs of words to study. They were told that they would later be given a cued recall test during which they would be shown one of the two words (bidirectional recall test). During the second phase participants solved simple arithmetic equations, which served as a distractor task. During the third phase participants were tested on cued recall for the 10 pairs from the study phase. Each session began with a short practice block to familiarize the participants with the experimental task. Each participant then studied and recalled 20 lists, with short breaks after every fifth list.

**Figure 1:**
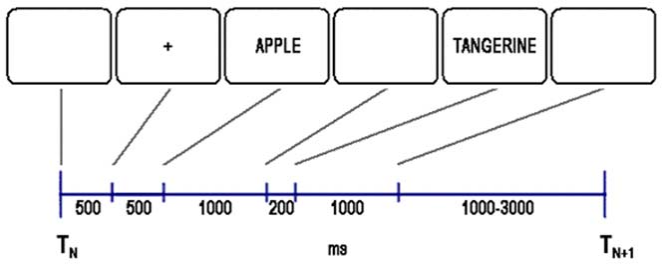
The time course for the evoked potential trial to the paired associates. T_N_ is the beginning of the Nth trial and T_N+1_ the beginning of the subsequent trial.

The evoked trial corresponded to the presentation of a pair of words during encoding. At the start of the evoked trial (Figure 1) a 500 ms delay was followed by central ‘+’ which served as a warning and lasted for 500 ms. The first word of a pair was then presented for 1000 ms, followed by a blank screen for 200 ms. The second word of a pair was then presented for 1000 ms. The inter-trial interval varied randomly between 1000 and 3000 ms.

After all 10 pairs had been presented, participants solved eight arithmetic problems of the form A+B+C = ? where A, B and C were randomly selected integers between 0 and 9. Each equation was presented on the screen for 3750 ms, followed by a 250 ms blank screen. Within this 4000 ms period, participants were asked to respond aloud, as quickly and accurately as possible. After giving their response, participants moved on to the next arithmetic problem without delay.

After participants finished solving the arithmetic equations, they were given a cued recall test for all the pairs that had been presented in the study phase of that cycle. During recall, a central ‘+’ was presented for 200 ms, followed by a cue word for 7000 ms. The first and second words served equally often as the cue. Participants responded vocally with the word they believed had been paired with the presented cue word. The experimenter scored the responses in real-time by referring to an answer key and pressing the “R” button of the keyboard for correct responses and the “N” button for incorrect responses. Incorrect responses and the absence of any response given by the participant within the allotted 7000 ms interval were classified as not-recalled. The next cue word was presented once the experimenter had coded the participant's response into the computer or when the time limit of 7000 ms was reached.

Individual participant waveforms were averaged based on subsequent memory performance, which resulted in two types of waveforms: one waveform corresponding to subsequently recalled pairs (R); and one waveform corresponding to subsequently not-recalled pairs (N). Next, both waveforms were broken down into sections corresponding to the presentation of Word1 and Word2. The ERP data recorded during the one second presentation of Word1 and the one second presentation of Word2 were both baseline corrected to each of the preceding 200 ms intervals. Then Word2-Word1 subtractions were derived separately for the R and N waveforms, resulting in two difference waveforms: the Word2-Word1 difference for R pairs [R(Word2−Word1)] and for N pairs [N(Word2−Word1)].

### Principal component analysis (PCA)

A standard PCA [28] was used to extract a reduced number of components, which revealed the spatial distribution of electrodes that displayed similar ERP patterns over time. The PCA was conducted with varimax rotation on the R(Word2−Word1)−N(Word2−Word1) difference. The input to the temporal PCA was the data matrix for the 65 electrode site variables by 250 time point observations (the sampling rate was 250 Hz and the dataset covered a 1000 ms interval) averaged across 12 participants. For each component, the corresponding factor scores were used to identify temporal addresses that showed a difference between the R(Word2−Word1) and N(Word2−Word1) waveforms. Representative electrodes were used to examine whether these differences could be attributable to amplitude differences between the ERPs to Word1 and Word2 of R and N pairs, as opposed to time shifts. Representative electrodes were those with large factor loadings and a central location within the cluster of electrodes that demonstrated the largest factor loadings. A repeated measures ANOVA was conducted to examine components of interest. Subsequent memory performance (R vs. N), word order within the pair (Word1 vs. Word2), and electrode location were included in the analysis as factors, and all interactions between these factors were also examined. The ANOVA was conducted on the data recorded from six representative electrodes. Unless specified as otherwise, significance testing was conducted over a ±25 ms range around the peak identified in the principal component wave.

## Results

As reported in our previous paper [21], subsequent memory performance was calculated as a function of intra-list context condition. The percentage of paired associate recall was higher for the condition that had low intra-list semantic similarity (*M = *70.9, *SE = *3.1) compared with the condition that had high intra-list semantic similarity (*M = *42.4, *SE = *3.4; t(11) = 9.70, p<0.001). An evaluation of recall performance as a function of serial position showed a borderline main effect of serial position [F(9, 111) = 1.99, p = 0.046]. However, post hoc comparisons indicated no significant differences between positions other than between serial positions 3 and 9 (p = 0.04).

**Figure 2:**
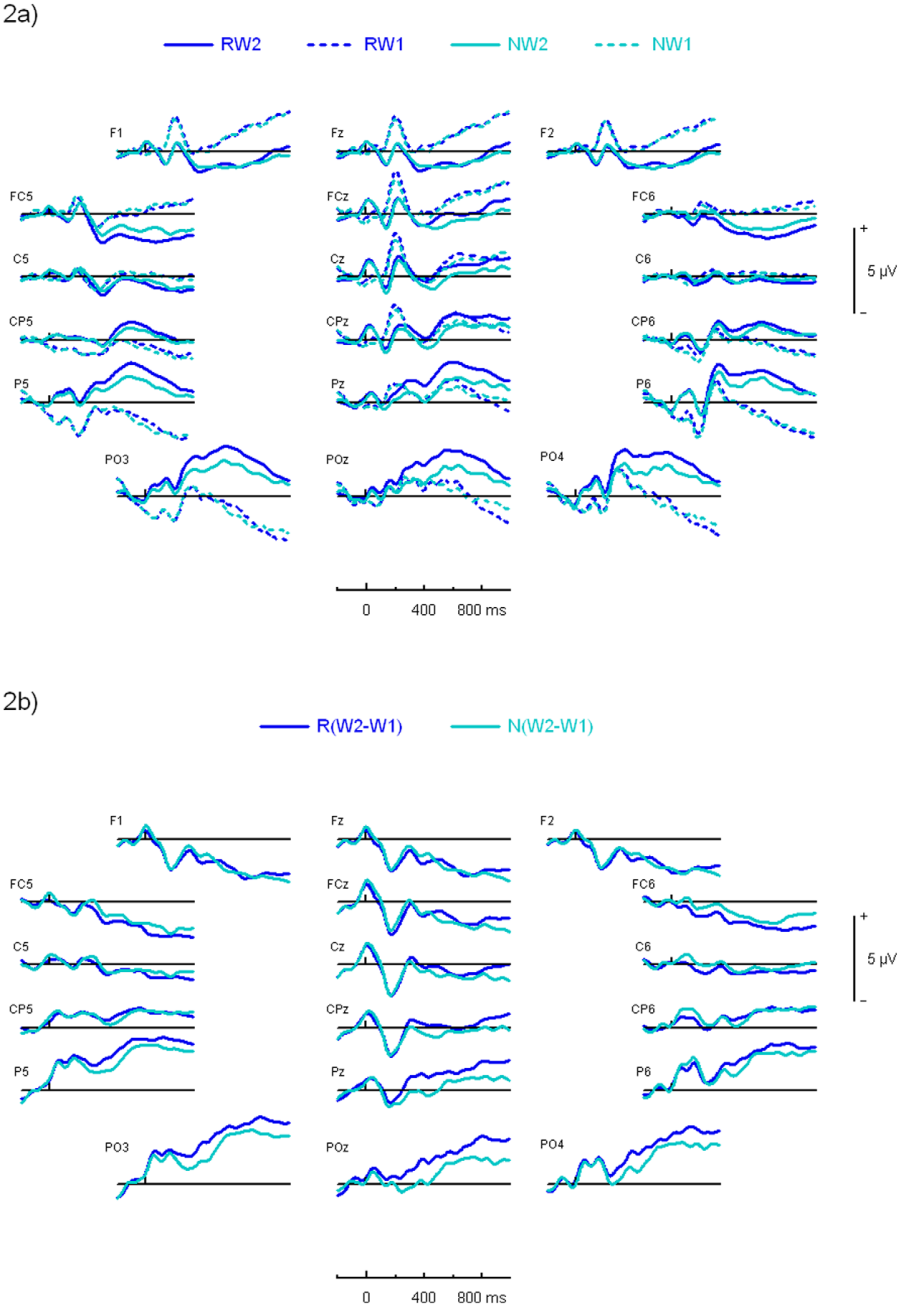
Grand average study phase ERP waveforms. a) Grand averages for ERPs to the first word of subsequently not-recalled pairs (NW1), to the second word of subsequently not-recalled pairs (NW2), to the first word of subsequently recalled pairs (RW1), to the second word of subsequently recalled pairs (RW2); 2b) Word2−Word1 difference waves for subsequently recalled pairs [R(W2−W1)] and subsequently not-recalled pairs [N(W2−W1)] at the group level.

Differences between the R(Word2−Word1) and N(Word2−Word1) waveforms were largest over the parietal region of the scalp. Waveforms recorded at multiple electrodes over the frontal, central and parietal scalp regions are shown in figure 2a. Over the parietal region of the scalp, the amplitude of the R(Word2−Word1) wave was more positive than the N(Word2−Word1) wave starting at around 200 ms, and continuing through to the end of the 1000 ms period (figure 2b). Figure 2a shows that this difference in amplitude between the R(Word2−Word1) and N(Word2−Word1) waveforms was mainly attributable to differences in the amplitude of the ERPs to Word1 and Word2 of the R pairs, whereas the amplitude of the ERPs to Word1 and Word2 of the N pairs did not appear to differ. The N(Word2−Word1) waveform was then subtracted from the R(Word2−Word1) waveform, resulting in the double difference waveform mentioned above.

### Principal component analysis

The PCA on the R(Word2−Word1)−N(Word2−Word1) difference resulted in a six-component solution when the eigenvalue threshold was set to one. The resulting six-component solution accounted for 97% of the total variance in the dataset. Components five and six together accounted for 6% of the total variance. These two components were discarded because there were very few electrodes loading onto these components, and we considered it plausible that the components were serving only to explain random noise detected by these electrodes. The fourth component accounted for 8% of the total variance and was also discarded because the corresponding waveform did not show a clear pattern. The topographic distributions of the first three components were substantially unchanged when the analysis was restricted to a three component solution. The three-component solution accounted for 86% of the total variance: the first principal component accounted for 55% of variance, the second principal component accounted for 17% of variance, and the third principal component accounted for 14% of variance. The first principal component (PC1) was of chief interest, because it provided evidence that was directly relevant to the purpose of the present study, and will be described in more detail below.

**Figure 3:**
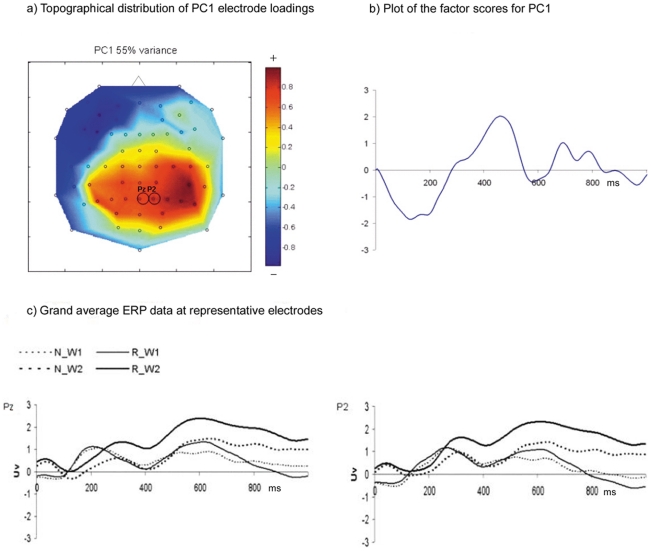
Results of the principal component analysis. a) The topographical distribution of electrode loadings from the rotated component matrix for the first principal component (PC1). The top of the figure corresponds to the front of the head; b) A plot of the factor scores for PC1; c) Grand average ERP data at representative electrodes Pz and P2 for PC1. These representative electrodes are circled in figure 3a. N_W1 = first word of subsequently not-recalled pairs; N_W2 = second word of subsequently not-recalled pairs; R_W1 = first word of subsequently recalled pairs; R_W2 = second word of subsequently recalled pairs.

The pattern of the factor loadings for PC1 (55% of variance) of the R(Word2−Word1)−N(Word2−Word1) difference was most salient over the posterior scalp region, as shown by the topographical distribution of the electrode loadings in figure 3a. The pattern of the PC1 factor scores demonstrated a negative deflection at about 130 ms (N130), followed by a positive deflection at about 460 ms (P460) and sustained positivity between 645–845 ms, as shown in figure 3b. The grand average ERP waveforms for representative electrodes Pz and P2 (figure 3c) showed that the N130 was due to a larger difference between the amplitude of ERPs to Word1 and Word2 of N pairs compared to R pairs. The P460 appeared to be due to an amplitude difference between ERPs to Word1 and Word2 of R pairs, with no apparent difference between the amplitude of ERPs to Word1 and Word2 of N pairs and ERPs to Word1 of R pairs. The data recorded at electrodes Pz and P2 also showed that the sustained positivity that was picked up by PC1 between 645–845 ms was due to larger amplitude differences between the ERPs to Word1 and Word2 of R pairs compared to N pairs.

A repeated-measures ANOVA was conducted on the N130, P460 and the sustained positivity observed between 645–845 ms, which will hereafter be referred to as the positive slow wave, to test whether the amplitude of these components demonstrated an interaction between subsequent memory performance and word order. The ANOVA was conducted using the data recorded at electrodes CPz, CP2, Pz, P2, P4, and P6. The full results of the ANOVA are listed in table 1. Here, we report on the interaction between subsequent memory performance and word order, as it is the result of interest for the purpose of the present study. The P460 demonstrated an interaction between subsequent memory performance and word order [F(1, 11) = 8.97, p = 0.01, partial eta-squared = 0.45]: the ERPs to Word2 were more positive than the ERPs to Word1 for the R pairs [*t*(71) = −2.631, p = 0.01], but there was no such difference for the N pairs. For the positive slow wave, the results of the repeated-measures ANOVA also showed an interaction between subsequent memory performance and word order [F(1, 11) = 10.11, p = 0.009, partial eta-squared = 0.48]. For the subsequently recalled pairs, the amplitude for Word2 was more positive than the amplitude for Word1 [*t*(71) = −4.38, p<0.001]. In contrast, the amplitudes for Word1 and Word2 did not differ for the subsequently not-recalled pairs. The N130 did not demonstrate an interaction between subsequent memory performance and word order.

**Table 1:** Results of the repeated measures ANOVAs for the first principal component (PC1).

Sources of Variance	Statistic	PC1: N130	PC1: P460	PC1: 645–845 ms
Subsequent Memory	F	1.151	13.647	8.904
	p	0.31	0.004	0.01
Word Order	F	0.329	0.151	2.697
	p	0.58	0.71	0.13
Electrode	F	0.737	4.925	11.241
	p	0.599	0.001	<.001
Subsequent Memory	F	0.108	8.968	10.112
X Word Order	p	0.75	0.01	0.009
Subsequent Memory	F	0.116	0.655	1.541
X Electrode	p	0.99	0.66	0.19
Word Order X Electrode	F	12.173	11.108	19.112
	p	<.001	<.001	<.001
Subsequent Memory X	F	3.090	1.470	2.220
Word Order X Electrode	p	0.02	0.22	0.07

## Discussion

The present study extends our previous investigation of cognitive association formation [21], by differentiating the electrical brain activity underlying item encoding and association formation. ERP components reflecting association formation were expected to occur after the presentation of the second word of a pair, based on the assumption that both words must be perceived before an association can be formed between the specified words. The data most relevant to the purpose of the present study consist of those encoding-related ERP components that showed significant amplitude differences between the first and second words of those pairs that were subsequently recalled and no significant amplitude differences between the words of those pairs that were not subsequently recalled. Two such ERP findings were observed in the present study: the P460 and the positive slow wave, both of which were largest over the parietal scalp region and discussed further below. Based on the results of the present study alone, it is unclear whether the observed P460 and positive slow wave correspond to the same or different cognitive processes. Our interpretations of these ERP components are based on the observed findings combined with the results of past studies. Further experiments will have to be conducted to determine whether these two ERP components index the same or different cognitive processes.

Interestingly, positive slow waves occurring over the parietal region have been associated with the completion of a task that is prompted by target detection [29], [30]. The slow wave is preceded by the P300 wave, which has been related to stimulus evaluation [31]. To examine the generality of the cognitive processes that parietal slow waves reflect, Garcia-Larrea and Cezanne-Bert [32] investigated whether parietal slow waves could be dissociated from the preparation or execution of a motor response, updating of working memory, and response selection. To do so, these investigators used a paradigm that consisted of two tasks. The first task required participants to detect a target, which then prompted them to perform a second task that varied between the experimental conditions. The results of the study suggests that parietal slow wave positivities are related to the number of items retrieved from working memory, and can be dissociated from processes related to motor response and response selection. In the context of the present study, the observed positive slow wave may partially reflect the retrieval of the first and second words of a pair from working memory. However, given that our ERP contrast differentiated those pairs that were subsequently recalled from those that were not, in addition to differentiating ERPs elicited by the first and second words of a pair, the observed slow wave also likely reflects processes that are supplementary to retrieval from working memory. These supplementary processes likely reflect processes related to association formation.

In a study by Caplan and colleagues [23], where the neuroelectric correlates of association formation and list learning were differentiated, as described above, the results of a multivariate analysis showed a latent variable reflecting a significant SME for pairs, but not lists, which was prominent over posterior electrode sites and somewhat left-lateralized. The timing of the latent variable overlapped primarily with the time window of the slow wave, but also showed some overlap with the early and late positive components. These results for association formation are consistent with the findings of the present study – the observed P460 and positive slow wave, which are thought to reflect association formation but not the encoding of the individual items that make up a pair.

In light of previous work demonstrating interdependence between encoding and retrieval processes and representations [33]–[37], ERP patterns reflecting retrieval of associative information may also reveal clues about the neuroelectric correlates of cognitive association formation. The parietal old/new effect is thought to reflect recollection [26], [27], and more specifically, has been associated with recollection of associative information [38], [39]. Interestingly, the latency of the positive slow wave observed in the present study falls into the general time range of the parietal old/new effect and its topographical distribution is similar to the topographical distribution shown by Yu and Rugg [40] for an ERP contrast that narrowed in on recollection (‘recollected’ versus ‘confidently old’ judgments) between 500 to 800 ms after stimulus onset. Similarly, the results of a study by Woodruff and colleagues [41] also showed a larger positive deflection in the ERPs elicited by recollected, compared to confidently recognized, items between 500 to 800 ms over both the right and left hemispheres of the parietal scalp region. The similarities between the observed positive slow wave and the parietal old/new effect, in terms of latency and topographical distribution, suggest that similar cognitive and brain processes are engaged during encoding and retrieval of content-specific information that allow one to recollect a previously experienced event, and provides further support for the notion that processes and representations that are active during encoding are reinstated during successful retrieval. In the context of the present study, participants may have performed study-phase retrieval while they were encoding the pairs of words. The processes engaged during study-phase retrieval may have then been reinstated during retrieval that occurred in the test phase, which would help explain the correspondence observed between the encoding-related data of the present study and the retrieval data of the studies discussed above [40], [41].

Duzel and colleagues [42] have examined ERPs recorded from an amnesic patient, with damage that appeared to be isolated to the hippocampus. Interestingly, the investigators did not find a parietal old/new effect in the patient's ERPs that were recorded during recognition. They did, however, find an index of familiarity in the ERP data. These findings suggest that recollection, compared to familiarity, is more dependent on the hippocampal formation and further highlights the importance of this brain region to successful cortical reinstatement. In a model that integrates the perspectives of cortical reinstatement with complementary cognitive perspectives, including encoding specificity [37] and transfer-appropriate processing [34], Rugg and colleagues [25] identified the hippocampus to be of central importance. In this model the hippocampus has the role of encoding, storing and reinstating patterns of brain activity elicited by a stimulus event. Interestingly, Ranganath and colleagues [43] have shown that the activity of the hippocampus measured during encoding, in addition to that of the posterior parahippocampal cortex, is predictive of recollection-based memory performance. In contrast, the activity of the rhinal cortex, as measured during encoding, is predictive of familiarity-based recognition. It will be interesting to learn what future research will reveal about the specific cognitive and brain processes involved in both encoding and retrieval, as well as the resulting clinical applications.

The present study is based on the logic that participants cannot begin to form associations between pairs of items until both items have been presented. Participants may, however, begin preparing to make an association before the presentation of a pair is completed. For example, in the present study, participants may have started preparing to form an association after the first word of a pair was presented. According to the conceptual peg hypothesis [44], [45], the first word of a pair may have been used as a peg upon which the second word was integrated to form an association. According to this hypothesis, concrete nouns, compared to abstract nouns, serve more effectively as conceptual pegs because they are more conducive to imagery, which the hypothesis regards as a mediator of recall. Furthermore, the conceptual peg hypothesis suggests that as long as one word of a pair is highly imageable, a holistic association can be formed by integrating the remaining word, regardless of whether it is concrete or abstract, into the image generated for the peg. Pairs composed of two low-imageability words, however, cannot form a unified whole. Contrary to this notion, Madan and colleagues [46] have shown that pairs composed of two low-imageability nouns remained generally as holistic as pairs composed of two high-imageability words and pairs composed of both high-imageability and low-imageability words, even though they did not include a high-imageability word that could be used as a conceptual peg. Alternatively, since both words of a pair belonged to the same semantic category in the present study, participants may have started preparing to make an association by anticipating that the second word would belong to the same semantic category as the first.

Participants in the present study may have been better positioned to encode the first word of a pair more effectively compared to the second word. The same or similar processes underlying primacy effects shown in list learning studies [47], [48] could also apply to sequentially presented pairs of items, which would be consistent with explanations at the neuronal level for primacy effects [49]–[52]. Also consistent with this notion, Caplan's Isolation Principle [53] posits that paired associate and serial learning are ends of a continuum rather than distinct types of information. This principle proposes that pairs of consecutive items are relatively isolated from other study items in paired associated learning paradigms (e.g., the interval separating two items of a pair is typically shorter than the interval separating consecutive pairs) but not in serial list learning paradigms, resulting in differential interference that can account for the nearly perfect correlation between forward and backward probes of pairs compared to the moderate correlation for serial lists. In line with the Isolation Principle, associative chaining models build serial lists by making associative links between consecutive items [54]. Interestingly, a recent study [55] investigated memory for within-pair order by examining the relation between forward and backward probes of pairs subject to order dependent associative interference and found that within-pair order is neither perfect as predicted by matrix models of memory, nor poor as predicted by convolution-based models that assume that within-pair order is not explicitly stored. The investigators of this study suggested that memory for within-pair order in verbal paired associate learning paradigms are supported by a mechanism vulnerable to error, thus, any model of paired associate learning must incorporate an assumption that within-pair order encoding is unreliable.

In summary, the results of the present study extend those reported by previous ERP studies on cognitive association formation, by differentiating the electrical brain activity underlying the encoding of individual words and associations formed between semantically related words. The positive slow wave observed in the present study likely reflects brain responses underlying the formation of associative bonds between the first and second words of a pair. The observed P460, on the other hand, likely reflects brain responses underlying the processing of the second word as the completion of the pair, which is regarded as being necessary for association formation to occur, and may have lead to the positive slow wave that followed. The results of the present study provide neuroelectric evidence that suggests different memory processes underlie item encoding and association formation.
